# Copper (II) Metallodendrimers Combined with Pro-Apoptotic siRNAs as a Promising Strategy Against Breast Cancer Cells

**DOI:** 10.3390/pharmaceutics12080727

**Published:** 2020-08-02

**Authors:** Natalia Sanz del Olmo, Marcin Holota, Sylwia Michlewska, Rafael Gómez, Paula Ortega, Maksim Ionov, Francisco Javier de la Mata, Maria Bryszewska

**Affiliations:** 1 Department of Organic Chemistry and Inorganic Chemistry and Research Institute in Chemistry “Andrés M. del Río” (IQAR), University of Alcala, 28805 Madrid, Spain; n.sanzdelolmo@gmail.com (N.S.d.O.); rafael.gomez@uah.es (R.G.); javier.delamata@uah.es (F.J.d.l.M.); 2 Networking Research Center on Bioengineering, Biomaterials and Nanomedicine (CIBER-BBN), Spain and Institute “Ramón y Cajal” for Health Research (IRYCIS), 28029 Madrid, Spain; 3 Department of General Biophysics, Faculty of Biology & Environmental Protection, University of Lodz, Pomorska 141/143, 90–236 Lodz, Poland; marcin.holota@op.pl (M.H.); maria.bryszewska@biol.uni.lodz.pl (M.B.); 4 Laboratory of Microscopic Imaging & Specialized Biological Techniques, Faculty of Biology & Environmental Protection, University of Lodz, Banacha12/16, 90–237 Lodz, Poland; sylwia.michlewska@biol.uni.lodz.pl

**Keywords:** Pro-apoptotic siRNA, copper, dendrimers, delivery vectors, in vitro, anticancer activity

## Abstract

Cancer treatment with small interfering RNA (siRNA) is one of the most promising new strategies; however, transfection systems that increase its bioavailability and ensure its delivery to the target cell are necessary. Transfection systems may be just vehicular or could contain fragments with anticancer activity that achieves a synergistic effect with siRNA. Cationic carbosilane dendrimers have proved to be powerful tools as non-viral vectors for siRNA in cancer treatment, and their activity might be potentiated by the inclusion of metallic complexes in its dendritic structure. We have herein explored the interaction between Schiff-base carbosilane copper (II) metallodendrimers, and pro-apoptotic siRNAs. The nanocomplexes formed by metallodendrimers and different siRNA have been examined for their zeta potential and size, and by transmission electron microscopy, fluorescence polarisation, circular dichroism, and electrophoresis. The internalisation of dendriplexes has been estimated by flow cytometry and confocal microscopy in a human breast cancer cell line (MCF-7), following the ability of these metallodendrimers to deliver the siRNA into the cell. Finally, in vitro cell viability experiments have indicated effective interactions between Cu (II) dendrimers and pro-apoptotic siRNAs: Mcl-1 and Bcl-2 in breast cancer cells. Combination of the first-generation derivatives with chloride counterions and with siRNA increases the anticancer activity of the dendriplex constructs and makes them a promising non-viral vector.

## 1. Introduction

New therapeutic approaches for cancer treatment are constantly being researched, especially where the side-effects of current therapies can be minimised. Within the new lines of research, suppressing the expression of specific genes is of particular importance in processes of cell development and differentiation in cancer and defence against viruses. RNA interference currently seems to be a growing technique in molecular biology due to its high potential in terms of therapeutic value [1]. In this sense, siRNA is the subject of clinical research, with encouraging results in the treatment of viral infections, neurodegenerative diseases, and cancer [2,3]. siRNA is formed by 2-stranded RNA molecules of approximately 20 nucleotides in length, whose strands complement each other perfectly. The mechanism of action of siRNA begins when the ‘antisense’ strand assembles into the RNA-induced silencing complex (RISC), which is used to identify the complementary messenger RNA. RISC then catalyses the cutting of the mRNA into halves, which are degraded by cellular mechanisms, thereby blocking the expression of the gene [4,5].

However, RNAi-based therapies are not without technical challenges, and the efficacy of their activity is conditioned due to their rapid degradation by nucleases, as well as to their negatively charged molecules being unable to easily penetrate the lipid cell membranes without a carrier to assist them. Many studies now focus on the use of a wide variety of nanoparticles as carriers for nucleic acids, such as inorganic or polymeric nanoparticles, liposomes, and dendrimers [6,7,8,9]. Furthermore, the nanocarriers have the potential to improve the pharmacokinetics and biodistribution of siRNA significantly and can be chemically modified to anchor another bioactive molecule into its structure to implement its therapeutic action for which the siRNA has been designed. For example, mesoporous silica nanoparticles that incorporate folic acid promoting endocytosis [10] or amphiphilic dendrimer with RGDK peptide sequences [11] have been described as efficient vectors for transfection of various siRNAs. Dendrimers are monodisperse materials that have interesting properties that can be used in different biomedical fields and have proved useful transfecting agent [12]. The possibility of incorporating different therapeutic fragments in a controlled way to dendritic scaffold opens the door to use heterofunctional systems with different modes of action. In the case of cancer, combination of siRNA with anticancer organometallic compounds supported on dendritic systems is a new and powerful therapeutic approach to potentiate their activity [13].

Organometallic complexes of platinum, ruthenium, silver, and copper, among others, have proven to be excellent anticancer agents; many of them have been introduced in different nanoparticles or dendritic systems to increase their bioavailability and improve their therapeutic action [14,15,16]. Some of them, such as cationic ruthenium carbosilane dendrimers [13] or multifunctional selenium nanoparticles [17] used as siRNA carriers, have a synergistic effect through their combined action. In the search for other alternatives among different transition metals, copper is emerging as a good candidate for the development of drugs for the treatment of cancer. This is a consequence due to its rich redox chemistry propitiated by its two states of oxidation, giving rise to the generation of reactive oxygen species (ROS) and excellent properties as an anti-cancer agent, along with high biocompatibility [18,19]. The mechanism by which the different copper (II) complexes exert their anti-cancer activity is still unknown. Furthermore, copper compounds are able to induce apoptosis in cancer cells [15]. Our research group has now shown how the inclusion of copper (II) complex into the carbosilane dendritic skeleton potentiated its anticancer activity in different cancer cell lines (PC-3, HL-60, HeLa), with promising results from both in vitro and in vivo experiments [15,20]. Copper (II) carbosilane dendrimers have shown an extraordinary interaction with different model cell membranes, such as lecithin liposomes and CTAB micelles [21].

The objective of our work has been to study the capacity of copper (II) carbosilane dendrimers as transfecting agents of pro-apoptotic siRNA, and to assess any cooperative effect between both fragments showing anticancer activity. For this purpose, we selected two different anticancer siRNAs—Mcl-1 (Myeloid cell leukaemia-1) and Bcl-2 (B-cell lymphoma 2)—which play a crucial role in the regulatory genes of apoptosis [22,23]. Herein, we describe the biophysical characterisation of complexes formed between copper (II) carbosilane dendrimers (G_n_-[Cu]) and pro-apoptotic siRNA, their ability to internalise the siRNA into cancer cells, and the cytotoxicity in human breast cancer cell line (MCF-7) in which Mcl-1 and Bcl-2 are overexpressed [22,24].

## 2. Materials and Methods

### 2.1. General Considerations

**Figure 1:**
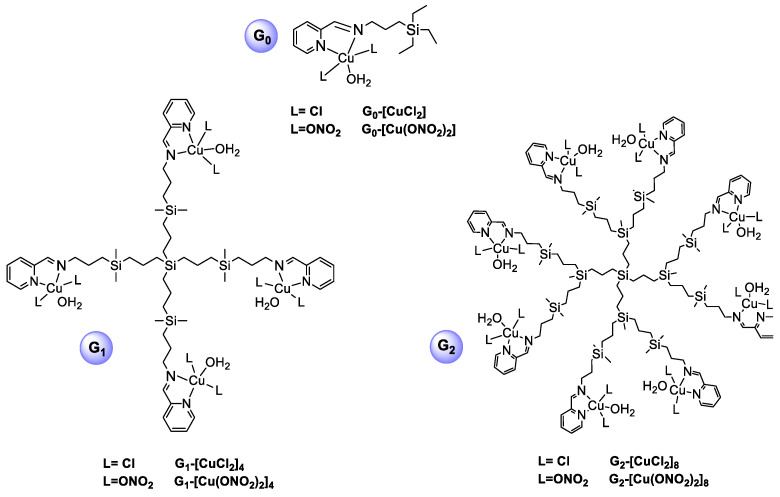
Chemical representation and nomenclature to the Schiff-base copper (II) metallodendrimers of zero (G_0_), first (G_1_) and second (G_2_) generation.

Copper(II) metallodendrimers: 2 groups have been investigated: Schiff-base copper (II) carbosilane metallodendrimers comprising chloride G_n_-[CuCl_2_]_m_ (n = 0, m = 1; n = 1, m = 4; n = 2, m = 8), and nitrate G_n_-[Cu(ONO_2_)_2_]_m_ (n = 0, m = 1; n = 1, m = 4; n = 2, m = 8) as counterions previously reported [18] (Figure 1).

siRNA structures: 2 different anticancer siRNAs—Mcl-1 (Myeloid cell leukaemia-1) and Bcl-2 (B-cell lymphoma 2)-were synthesised (Darmacon, Inc., Lafayette, CO, USA). The experiments were carried out with the fluorescein-labelled siRNAs for electrophoresis, fluorescence measurements, and internalisation techniques. Sense and antisense sequences are included in Table 1.

### 2.2. Biophysical Evaluation of the Dendriplexes

#### 2.2.1. Gel Electrophoresis

[siRNA] = 2 µM, G_n_-[Cu]/siRNA complexes were prepared at pH 7.4 in 10 mM phosphate-buffered saline. G_n_-[Cu]/siRNA molar ratios ranged from 1:1 to 1:100. Electrophoretic conditions: 3% agarose gel with GelRed stain was run in Tris-acetate-EDTA (TAE) buffer for 45 min at 90 V/35 mA and detected with UV Protective assays. G_n_-[Cu]/siRNA was incubated with 3 µg/mL RNase at 37 °C for 30 min. The samples were incubated on ice for 10 min before 0.082 mg/mL heparin was added.

#### 2.2.2. Transmission Electron Microscopy (TEM)

10 μL of dendriplex solutions (molar ratio [G_n_-[Cu]]/[siRNA] = 30) were coated on 200-mesh copper grids with carbon surface after 15 min incubation. The samples were stained with uranyl acetate solution for 20 min. The grids were washed with deionised water and dried at room temperature before examination in a JEOL-1010 (JEOL, Tokyo, Japan) transmission electron microscope.

#### 2.2.3. Zeta Potential Measurements

Zeta potential values were measured using a Laser Doppler Velocimetry technique by Zetasizer Nano ZS-90, Malvern Instruments (UK) and calculated using the Helmholtz-Smoluchowski equation. Conditions: [siRNA] = 0.3 μM, [G_n_-[Cu]]/[siRNA] molar ratios ranged from 0.5 to 50 with water as solvent. Analysis of data was carried out using Malvern software and given as mean ± standard deviation (SD) obtained by 7 measurements in 5 cycles at room temperature for each sample.

#### 2.2.4. Hydrodynamic Diameter of the Dendriplexes

Conditions: [siRNA] = 0.3 μM, [G_n_-[Cu]]/[siRNA] molar ratios ranged from 0.5 to 50 with distilled water as solvent. The Malvern Zetasizer was used to determine the hydrodynamic diameter of the dendriplexes by dynamic light scattering in the same manner as in 2.2.3.

#### 2.2.5. Fluorescence Polarisation Measurements

Fluorescence polarisation of labelled siRNA was measured with a PerkinElmer LS-50B spectrofluorimeter (Perkin-Elmer, Waltham, MA, USA). The results are shown as the ratio between sample and control values (r/r_0_). Conditions of measure: [siRNA] = 0.35 μM, complex formed at 37 °C, pH 7.4, in 10 mM Na-phosphate buffer at molar ratios [G_n_-[Cu]]/[siRNA] ranging from 0.5 to 50. Excitation wavelength of 485 nm was with an excitation-slit width set at 2.5 nm, and emission wavelength 516 nm with an emission-slit set of 3 nm. Data collected are mean ± standard deviation (SD) of a minimum of 3 independent experiments.

#### 2.2.6. Circular Dichroism

Circular dichroism (CD) measurements involved a Hellma quartz cells with a thickness of 0.5 cm in a J-815 CD spectrometer (Jasco, Tokyo, Japan), with software provided by Jasco being used to calculate the mean ellipticity values. Complex formation: on to 1 μM siRNA, different amounts of G_n_-[Cu] dendrimers were added in a 10 mM Na-phosphate buffer, pH 7.4, in a range of molar ratios [G_n_-[Cu]]/[siRNA] from 1 to 31. Wavelength was set from 235 to 300 nm, and the parameter assays were: 0.5 nm step resolution, 1.0 nm bandwidth, 4 s response time, and 50 nm/min scan speed. The slit was set on auto.

### 2.3. Evaluation of Anticancer In Vitro

#### 2.3.1. Cell Cultures

MCF-7, purchased from ATCC cell lines (Manassas, VA, USA), were grown in plastic tissue culture flasks (Falcon, GE Healthcare Life Sciences, Chicago, IL, USA) at 37 °C in a humidified air atmosphere with 5% CO_2_. DMEM (Gibco, Thermo Fisher Scientific, Waltham, MA, USA) was supplemented with 10% heat-inactivated fetal bovine serum (FBS, HyClone, GE Healthcare Life Sciences, Chicago, IL, USA) and 1% antibiotic (penicillin/streptomycin).

#### 2.3.2. Cytotoxicity

MTT (MTT, 3-(4,5dimethyl 2-thiazolyl)-2,5-diphenyl-2H-tetrazolium bromide) was measured. Cells were seeded on a 96-well plate at 1 × 10^4^ cells per well. After 72 h incubation with G_n_-[Cu] dendrimers and G_n_-[Cu]/siRNA complexes, MTT (0.5 mg/mL of MTT in PBS) was added for 3 h to allow formazan crystals to form, which were dissolved in DMSO. Optical density directly proportional to the surviving cells was measured at 580 nm (background correction at 720 nm) using a multiwell plate reader (BioTek PowerWave HT, BioTek Instruments, Inc. Winooski, VT, USA). Results were calculated from the following equation:

% viability = (A/Ac) × 100

where Ac, is the absorbance of the control cells (non-treated) and A is the absorbance of the sample. The results are given as mean ± standard deviation (SD) from 3 independent experiments.

#### 2.3.3. Statistical Analysis

The results were collected from a minimum of 3 independent experiments and given as mean ± standard deviation (SD). One-way analysis of variance (ANOVA) and Tukey’s test was applied. Significance was accepted at *p* ≤ 0.05 (*); *p* ≤ 0.01 (**); *p* ≤ 0.001 (***).

## 3. Results and Discussion

Two families of copper (II) metallodendrimers, (G_n_-[CuCl_2_]_m_ (n = 0, m = 1; n = 1, m = 4; n = 2, m = 8) and G_n_-[Cu(ONO_2_)_2_]_m_ (n = 0, m = 1; n = 1, m = 4; n = 2, m = 8) (Figure 1), were selected to assess the influence of dendrimer generation and the metal counter-ion (chloride and nitrate) on their ability to form complexes with siRNA and be delivered to cancer cells. These compounds had positive charges in zeta potential measurements, corroborating their ionic nature, with the positive charge on the metal complex and the negative charge on the counterion (chloride and nitrate) [15]. The siRNAs chosen to carry out the study were Mcl-1 and Bcl-2, which are overexpressed in MCF-7cells.

### 3.1. Evaluation of the Interaction Between Ru(II) Metallodendrimers and siRNA

#### Electrophoresis Assays

**Figure 2:**
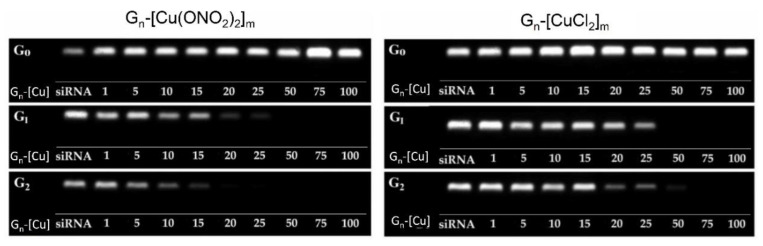
Formation of G_n_-[Cu]/siRNA complexes at different molar ratios. G_n_-[Cu]: dendrimer, siRNA naked [siRNA] = 2 µM, 1–100 dendriplexes migration at different molar ratios. Complexes prepared as described in the text were analysed by electrophoresis on 3% agarose gel with siRNA migration being identified by fluorescein-labelled siRNA.

Gel electrophoresis is commonly applied to characterise nanocomplex (also called dendriplex) formation and calculate [G_n_-[Cu]]/[siRNA] molar ratios. To visualise the dendriplex G_n_-[Cu]/siRNA formation, it is necessary to use a fluorescein-labelled Mcl-1 siRNA. Negatively charged siRNA can migrate freely in the gel, whereas the addition of positively charged Cu (II) metallodendrimers to the siRNA solution significantly delays siRNA migration, confirming the ability of copper metallodendrimers to complex with siRNA. To calculate the amount of dendrimers needed to complex a total of 2 μM siRNA, different molar ratios of G_n_-[Cu]/siRNA were used, ranging from 1 to 100. The electrophoregram shows that dendrimers of the first and second generation interacts with siRNA by forming positively charged complexes, whereas the dendrimers of zero generation do not retain the siRNA migration in the gel, probably due to their small size (Figure 2). It was also possible to observe different patterns of migration depending on the metal counterion (chloride and nitrate) in the dendrimer. Complexes formed with chloride counterion dendrimers (G_n_-[CuCl_2_]_m_) need molar ratios ranging between 1:25–1:50 to saturate the G_n_-[Cu]/siRNA, whereas nitrate complexes (G_n_-[Cu(ONO_2_)_2_]_m_) reach saturation at molar ratios ranging between 1:15–1:25 (Figure 2). This effect could be explained by the higher positive density of the charge of nitrate compounds, as the lability of the bond between the nitrate and the metal centre is higher than in chloride derivatives.

Once the capacity of Cu (II) metallodendrimers to form dendriplexes had been proven, one of the essential issues related to the efficient delivery of siRNA is its protection against digestion by nucleases. Based on the results obtained in a previous electrophoresis gel, we selected a molar ratio of G_n_-[Cu]/siRNA = 30 as the most suitable to assess protection against RNase-TEM assay, in vitro internalisation, and cell viability experiments.

**Figure 3:**
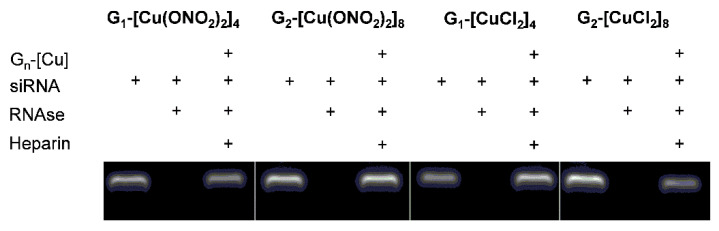
Protective effect of G_n_-[Cu(X)_2_]_m_ (X = Cl or ONO_2_) to siRNA against degradation by RNase. [siRNA]= 2 µM, [RNase A] =3.0 μg/mL for 30 min at 37 °C, [heparin] = 0.082 mg/mL.

As a control, naked siRNA incubated with RNAse showed no fluorescence signal in the gel that would indicate its degradation. Treatment of the G_n_-[Cu]/siRNA complexes with heparin after incubation with RNAse displaces the dendrimers from the complex, making it possible to observe the signal of undamaged siRNA in the agarose gel, thereby indicating the protective properties of all copper (II) metallodendrimers of both first and second generation (Figure 3).

### 3.2. Biophysical Characterisation of Dendriplexes

The dendrimers flexibility, as well as the nanoconjugates size, are determining factors in the transfection capacity; in general, higher dendritic flexibility and a smaller size are related to a more powerful transfection ability.

#### 3.2.1. Zeta Potential, the Hydrodynamic Diameter of Dendriplexes, and TEM Assays

**Figure 4:**
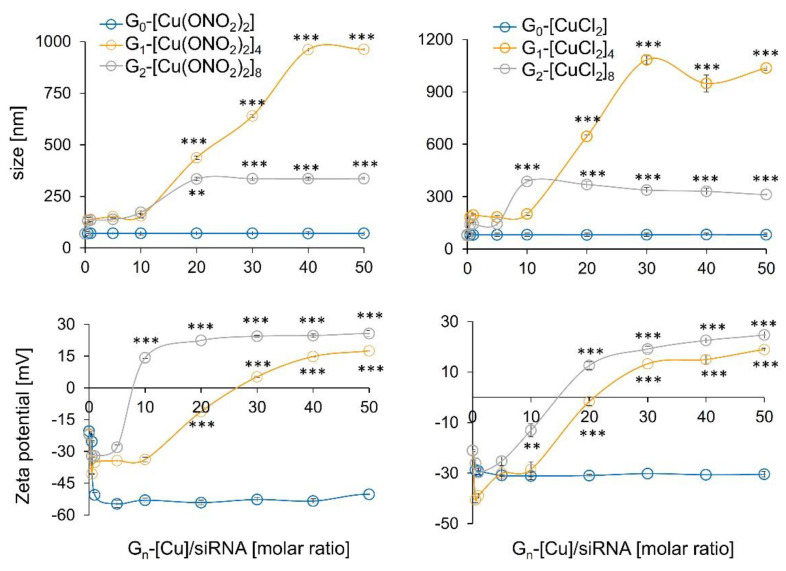
Zeta potential and zeta average size of G_n_-[Cu]/siRNA complexes at different molar ratios in water as a solvent. [siRNA] = 0.3 μM. Results were represented as mean ± standard deviation (n = 3). Statistically significant differences compared to the control cells *p* ≤ 0.01 (**); *p* ≤ 0.001 (***).

Zeta potential measurements determine the surface charges of complexes formed between the siRNA (negative zeta potential values) and dendritic systems. Addition of increasing amounts of first and second generation of cationic Cu (II) metallodendrimers induced a change towards positive values. In general, the lesser amount of the second-generation dendrimer compared to the first generation was needed to compensate the ionic charge, due to the high positive density of 8 positive charges against 4. Furthermore, as in electrophoresis assays, one could note a different behaviour depending on the counterion. Again, nitrate derivate dendrimers (G_n_-[Cu(ONO_2_)_2_]_m_) saturated the complex more quickly, with less concentration than chloride derivates (G_n_-[CuCl_2_]_m_), due to the nature of the metal-ligand bond. In contrast, the dendriplexes formed with the monometallic compound, regardless of the metal counter-ion present in the structure and the concentration used, did not increase the zeta potential values, indicating a lacking complexes formation (Figure 4).

**Figure 5:**
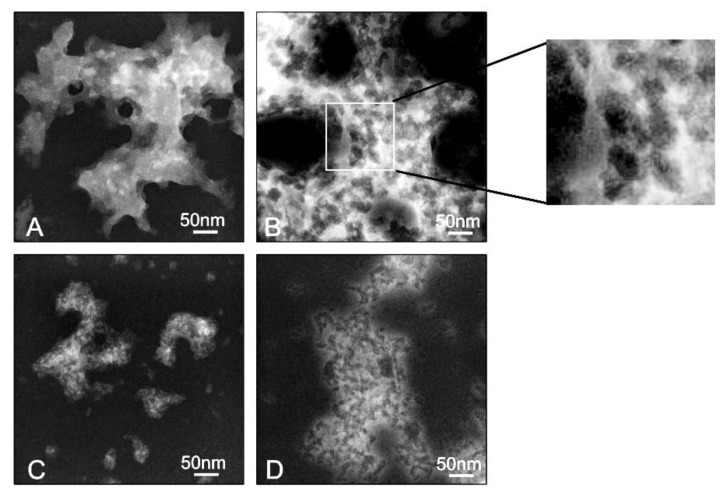
Ultrastructure of G_n_-[Cu]/siRNA complexes visualised by TEM: (**A**) G_1-_[Cu(ONO_2_)_2_]_4_, (**B**) G_2-_[Cu(ONO_2_)_2_]_8_ (**C**) G_1-_[CuCl_2_]_4_, (**D**) G_2-_[CuCl_2_]_8_.

The size and morphological structure of dendriplexes can be determined by using dynamic light scattering (DLS) to find their hydrodynamic diameter in solution and TEM for morphological structure, shape, and size. The DLS results show that those formed with dendrimers of the first generation were more prominent than those composed of the second generation, with sizes ranging from 980–1110 nm and 290–340 nm, respectively (Figure 4), and for the monometallic derivative, the size parameters were unchanged in respect to naked siRNA. These findings indicate the existence of aggregation between different dendriplexes confirmed by TEM, where, in all dendriplexes, visible aggregates with globular electron-dense structures inside were present (Figure 5). TEM images indicate that conjugates formed by G_1-_[CuCl_2_]_4_ are smaller in size (~50 nm), whereas other dendriplexes, G_1-_[Cu(ONO_2_)_2_]_4_, G_2-_[Cu(ONO_2_)_2_]_8_ and G_2-_[CuCl_2_]_8_, were of greater dimensions (~500 nm) (Figure 5). This phenomenon has been previously observed for other carbosilane systems of a cationic nature [25,26,27] or other dendritic skeletons as PAMAM [28,29] or phosphorus dendrimers [30]. The size and charge of the nanoparticle can be responsible for their interaction with immune cells [31]. Moreover, the larger nanoparticles (130 and 70 nm) were strongly affected by the drag force leading to the removal from endothelial cell surfaces [32]. Khopr et al. (2018) suggests that the anionic PISA nanoparticles (40 nm) seem to be good candidates for anti-cancer drug delivery while the bigger nanoparticles can be applied for the creation of vaccines and could be used as immunomodulators for B cells [32].

The difference in size found by DLS and TEM for the same dendritic structure can be explained by differences in sample preparation; while DLS is a technique carried out in solution, TEM looks at dry samples [4,33]. In all the cases analysed, there was also an effect on the hydrodynamic diameter of the complexes formed by siRNA and delivery systems, which was concentration-dependent, being higher with an increasing molar ratio.

#### 3.2.2. Fluorescence Polarisation and Circular Dichroism

**Figure 6:**
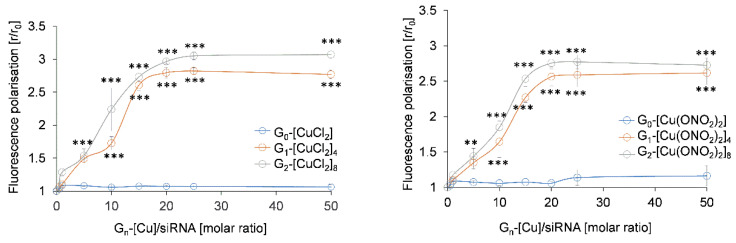
Changes in fluorescence polarisation of fluorescein-labelled Mcl-1 in the presence of increasing concentrations of different G_n_-[Cu] metallodendrimers. [siRNA] = 0.35 μM in Na-phosphate buffer 10 mM (pH 7.4). Results are represented as mean ± standard deviation (n = 3). Statistically significant differences compared to the control cells *p* ≤ 0.01 (**); *p* ≤ 0.001 (***).

The stability of dendriplexes was corroborated by fluorescence polarisation measurements of fluorescein-labelled Mcl-1. Changes in fluorescence polarisation of a probe attached to the siRNA structure can reflect an interaction between dendrimers and nucleic acids. Fluorescence polarisation decreased with first and second-generation dendrimers, whereas for monometallic ones, this was not observed (Figure 6). Plateau phase was reached at a molar ratio [G_n_-[Cu]]/[siRNA] = 20 for the dendrimers of both nitrate and chloride counterions. Increasing concentration of dendrimers of zero generation did not significantly change the level of fluorescence polarisation.

**Figure 7:**
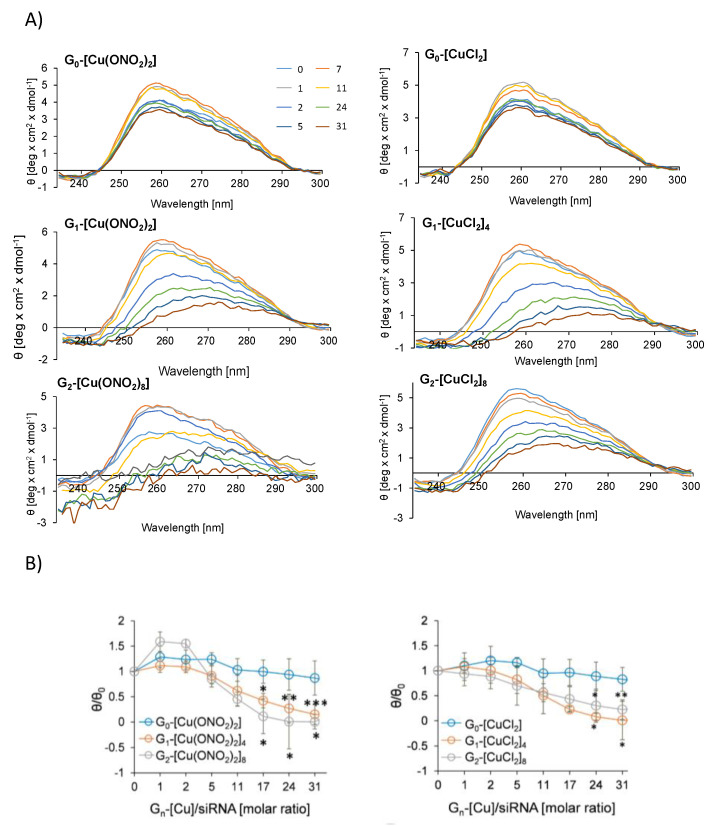
(**A**) CD spectra of siRNA at the presence of G_n_-[Cu] metallodendrimers in increasing molar ratios. G_n_-[Cu]/siRNA (**B**) Changes in the mean residue ellipticity (θ) of siRNA at λ = 258 nm in the presence of dendrimers. [siRNA] = 1 μM. Results are represented as mean ± standard deviation (SD), n = 3. Statistically significant differences compared to the control cells *p* ≤ 0.05 (*); *p* ≤ 0.01 (**); *p* ≤ 0.001 (***).

The influence of nanoconjugates on the secondary structure of siRNA was measured by circular dichroism [13,30]. The typical siRNAs CD spectra contains two characteristic peaks at λ = 210 and 260 nm. [7] However, we focused on the analysis of the ellipticity changes in the region of λ = 240–280 nm. A decrease in ellipticity (θ) in the range of a wavelength from 235 to 300 nm, accompanied by a reduction in the peak at 258 nm, was detected with increasing G_n_-[Cu]/siRNA molar ratios (Figure 7). CD spectra of siRNA in the presence of dendrimers reflected an alteration in the secondary structure of siRNA due to the binding of dendrimer molecules. The second-generation dendrimers showed the most intensive changes, whereas the monometallic derivatives do not significantly affect the structure of siRNA. Analysis of the changes in θ/θ_0_ parameter at 258 nm showed that at this wavelength, ellipticity values reached a plateau at a molar ratio G_n_-[Cu]/siRNA of 10 for G_1-_[Cu(ONO_2_)_2_]_4_, G_2-_[Cu(ONO_2_)_2_]_8_, G_1-_[CuCl_2_]_4_1 and 8 for G_2-_[CuCl_2_]_8_.

### 3.3. Biological Evaluation of CCD-siRNA Complexes: Cellular Uptake and Anticancer Activity

Cells treated with pro-apoptotic siRNA silences the expression of anti-apoptotic genes, which may result in the induction of apoptosis and, consequently, a decrease in cancer cell viability [4,34]. Cancer cells use different mechanisms to evade apoptosis and favour tumour progression. Family members of Bcl-2 proteins control mitochondrial-dependent apoptosis. High levels in these anti-apoptotic proteins, including Bcl-2 and Bcl-xL, prevent apoptosis and therefore impede cancer therapy. The influence of dendriplexes on MCF-7 involved the use of 2 different types of siRNAs, which were Mcl-1 and Bcl-2, leading to its overexpression. On the one hand, the involvement of Mcl-1 in breast cancer has been widely confirmed by the high basal levels of the mRNA found in the cancer compared to other subtypes. Moreover, it has become a target for breast cancer treatment and a crucial indicator in prognosis [22]. This human protein is encoded by the Mcl-1 gene that induces myeloid leukemia cell differentiation.

On the other hand, the Bcl-2 gene is overexpressed in 50–70% of breast cancer patients, giving rise to resistance to conventional treatments, making it a promising target. Silencing of this gene by siRNA in orthotopic xenograft models has shown that siRNA against Bcl-2 given intravenously significantly suppresses growth of MCF-7 cells. Moreover, this strategy significantly increases the efficacy when combined with doxorubicin [35].

#### 3.3.1. Cellular Uptake

**Figure 8:**
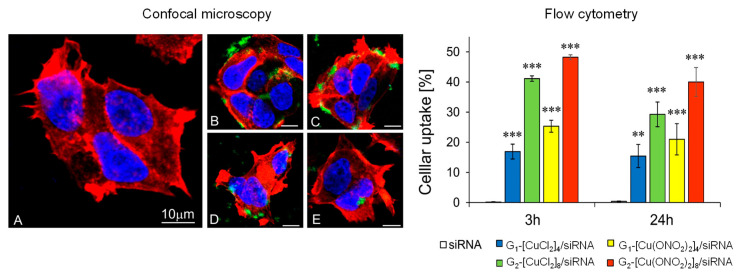
Confocal microscopy: (**A**) naked siRNA, (**B**) G_1-_[Cu(ONO_2_)_2_]_4_/siRNA (**C**), G_2-_[Cu(ONO_2_)_2_]_8_/siRNA (**D**) G_1-_[CuCl_2_]_4_/siRNA and (**E**) G_1-_[CuCl_2_]_4_/siRNA and flow cytometry data of uptake by MCF-7 cells. General conditions: incubation time: 3 h and 24 h; Bar = 10 µM; [siRNA] = 100 nM; molar ratio [G_n_-[Cu]/[siRNA]] = 30. The results are mean ± standard deviation (SD), n = 3. Statistically significant differences compared to the control cells *p* ≤ 0.01 (**); *p* ≤ 0.001 (***).

Confocal microscopy shows the ability of dendritic systems to transport siRNA into the cytoplasm, whereas naked siRNA does not cross the membrane without a transfection agent (Figure 8A). The results of confocal microscopy and flow cytometry techniques showed that the internalisation of the G_n_-[Cu]/siRNA complexes is more favourable after 3 h of incubation than after 24 h and is dependent on dendrimer generation and the nature of the counterion. The percentage of cell internalisation (≈50%) is raised after 3 h incubation with second-generation Cu(II) metallodendrimer with a nitrate counter-ions, G_2_-[Cu(ONO_2_)_2_]_8_, (Figure 8, bottom panel). In addition, the microphotographs show that Mcl-1 siRNA complexed with G_n_-[Cu(ONO_2_)_2_]_m_ dendrimers was more visible than siRNA complexed with G_n_-[CuCl_2_]_m_ dendrimers. Whereas G_n_-[Cu(ONO_2_)_2_]_m_ dendriplexes appeared inside cells as green dots, the complexes created with G_n_-[CuCl_2_]_m_ were mainly near the cell membranes, rarely in the cytoplasm (Figure 8, top panels). Although transfection will depend on the type of cell to be transfected, the applied dendrimer generation, and the time of incubation, we can say that copper (II) metallodendrimers, in general, have a greater transfection efficiency than the Ru(II) analogues in the HL60 a cell line (Ru(II)-second generation (≈30% efficiency) [12], and close to cationic carbosilane dendrimers with 16 positive charges on the surface [5]. The extension of incubation time up to 24 h can cause a gradual siRNA release from the complex, and, as a result, the siRNA interference phenomenon may be initiated [29].

#### 3.3.2. Anticancer Activity

**Figure 9:**
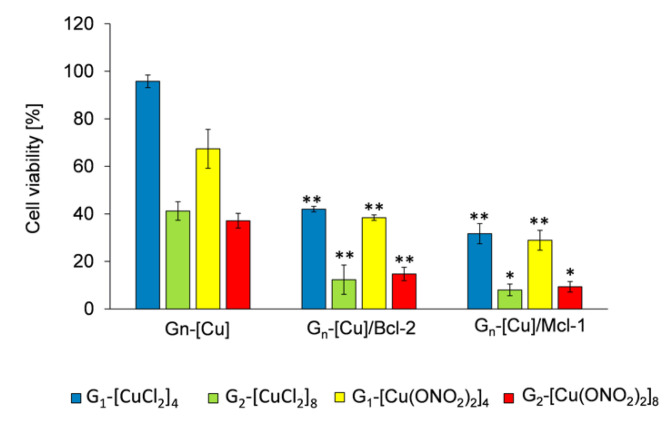
Viability of MCF-7 cells after 72 h of incubation with G_n_-[Cu] and their complexes with pro-apoptotic siRNAs (Mcl-1; Bcl-2) at molar ratio [G_n_-[Cu]]/[siRNA] = 30. Conditions: [G_n_-[Cu]] = 3 μM, [siRNAs] = 100 nM. Results are mean ± standard deviation (SD), n = 3. Statistically significant differences compared to the control cells *p* ≤ 0.05 (*); *p* ≤ 0.01 (**).

A possible cooperativity between Cu(II) metallodendrimers, which are active in a wide panel of cancer cell lines, and pro-apoptotic Mcl-1 or Bcl-2 siRNA, have been studied by MMT assay in MCF-7 cells after 72 h treatment. Previously, we reported the IC_50_ values for Cu(II)-systems in MCF-7 [21] and PBMC [15] cell line. The G_n_-[Cu] concentration chosen to carry out the viability assays here was a subtoxic concentration of 3 μM. Figure 8 shows the treatment of MFC-7 cells with dendrimer alone and dendriplex (molar ratio [G_n_-[Cu]/siRNA] = 30; G_n_-[Cu] = 3 μM and for siRNA = 100 μM) expressed as percentage viability. The results indicate that G_n_-[Cu]/siRNA complexes were more toxic to MCF-7 cells compared with uncomplexed dendrimers. As an example, MCF-7 treated with 3 μM G_1-_[CuCl_2_]_4_ had a viability of up to 95%, whereas dendriplexes G_1-_[CuCl_2_]_4/_Mcl-1 and G_1-_[CuCl_2_]_4_/Bcl-2 reduced viability to 32% and 43%, respectively (Figure 9). With second-generation systems, a reduction of ~90% in viability was seen when MFC-7 cells were treated with dendriplexes. This result shows a cooperative effect between fragments with anticancer activity and a different mode of action. In addition, regarding dendrimer size and nature of counterion, this finding shows that the dendritic generation affects the cytotoxicity of the dendriplexes, i.e., the first-generation dendrimers show the most promise due to the lack of toxicity when the dendrimers are administered alone. However, the presence of the different counterion (chloride or nitrate) did not appear to significantly affect the anticancer activity. Results previously obtained for other cationic carbosilane dendrimers of the second and third generations with 12 and 24 functional groups, respectively, in HeLa and HL-60 cell lines, showed that the combination of these dendrimers with si-Mcl-1, si-Bcl-2 and si-Bcl-xL, required a siRNA concentration of 250 nM and a molar ratio of [G_n_-[Cu]]/[siRNA] = 10 to have promising activity, despite the greater number of functional groups compared to copper metallodendrimers.

## 4. Conclusions

We have demonstrated the ability of cationic Cu (II) carbosilane dendrimers to transfect the pro-apoptotic siRNAs Mcl-1 and Bcl-2 and protect it against nuclease degradation. All the biophysical procedures carried out in this work indicate that monometallic derivatives under the applied conditions do not complex with siRNA, probably as a result of their small size, which does permit the creation of stable conjugates. In contrast, first- and second-generation copper (II) metallodendrimers not only formed dendriplexes with pro-apoptotic siRNA, but also protected them against degradation by nucleases. Moreover, Cu (II) metallodendrimers act as pure vehicles as well as enhancing the anticancer action of siRNA. To be highlighted is the fact that the first-generation dendrimer G_1_-[CuCl_2_]_4_ at 3 µM does not have a cytotoxic activity (viability 90%), but when combined with siRNAs, it can decrease viability by 35–40%, depending on the pro-apoptotic siRNA used. These findings show that copper (II) dendrimers can be powerful agents in cancer therapies as siRNA carriers or anticancer agents.

## Figures and Tables

**Table 1:** Sense and antisense sequences of two pro-apoptotic siRNAs (Mcl-1 and Bcl-2).

Strand	Mcl-1	Bcl-2
Sense	5′-GGACUUUUAUACCUGUUAUtt 3′	5′-G CUG CAC CUG ACG CCC UUCtt 3′
Antisense	5′-AUAACAGGUAUAAAAGUCCtg 3′	5′-GAA GGG CGU CAG GUG CAG Ctt 3′
